# Theoretical and Numerical Analysis of Mechanical Behaviors of a Metamaterial-Based Shape Memory Polymer Stent

**DOI:** 10.3390/polym12081784

**Published:** 2020-08-10

**Authors:** Ruoxuan Liu, Shuai Xu, Xiaoyu Luo, Zishun Liu

**Affiliations:** 1 International Center for Applied Mechanics, State Key Laboratory for Strength and Vibration of Mechanical Structures, Xi’an Jiaotong University, Xi’an 710049, China; liuruoxuan@stu.xjtu.edu.cn (R.L.); xushuai9776@xjtu.edu.cn (S.X.); 2 School of Mathematics and Statistics, University of Glasgow, Glasgow G12 8SQ, UK; xiaoyu.luo@glasgow.ac.uk

**Keywords:** mechanical behavior, metamaterial, radial strength, shape memory polymer stent

## Abstract

Shape memory polymers (SMPs) have gained much attention in biomedical fields due to their good biocompatibility and biodegradability. Researches have validated the feasibility of shape memory polymer stent in treatment of vascular blockage. Nevertheless, the actual application of SMP stents is still in infancy. To improve the mechanical performance of SMP stent, a new geometric model based on metamaterial is proposed in this study. To verify the feasibility and mechanical behavior of this type of stent, buckling analysis, and in vivo expansion performance of SMP stent are simulated. Numerical results exhibit that stent of a smaller radius behaves a higher critical buckling load and smaller buckling displacement. Besides, a smaller contact area with vessel and smaller implanted stress are observed compared with traditional stents. This suggests that this SMP stent attributes to a reduced vascular restenosis. To characterize the radial strength of SMP stent, an analytical solution is derived by the assumption that the deformation of stent is mainly composed of bending and stretch. The radial strength of SMP stent is assessed in form of radial force. Analytical results reveal that radial strength is depended on the radius of stent and periodic numbers of unit cell in circumferential direction.

## 1. Introduction

As a kind of smart material, shape memory polymers (SMPs) can recover to their original shape from the deformed state spontaneously with appropriate stimuli, such as heat [1,2], light [3], water [4], and so on. SMPs exhibit advantageous properties in large deformation, biocompatibility, and biodegradability, therefore showing promising applications in biomedical fields [5,6], such as scaffolds [7], and tissue engineering [8]. In particular, shape memory polymer stents are shown to reduce the restenosis of a vessel and have stable expansions of the plaque [9,10]. The feasibility of SMPs in vessel stents has already been validated in experiments [11] and numerical computations [12,13]. Wache et al. proposed the new concept of a SMP stent in drug delivery application [14]. Based on the highly controlled and tailored deployment of SMP, Baer et al. fabricated the light-activated SMP stent by thermoplastic polyurethane [15,16]. Their in vitro experimental results showed a full recovery ratio of a SMP stent. Yackacki et al. synthesized SMP networks and covered the effects of perforations and packaging temperatures on the recovery of a SMP stent [17]. The study was conducted to repair larger vessels such as abdominal aorta. In a numerical study, Reese et al. derived a new constitutive model of large strain and applied the model to describe the thermos-mechanical behavior of SMP stent [12]. Kim et al. investigated the feasibility of a shape memory polyurethane (SMPU) stent with a braided structure [18]. The finite element results showed a comfortable expansion of the SMPU stent. Furthermore, the specific expansion behavior was conducted by Liu et al. [13]. The work presented a tunable expansion performance of a SMP stent with recovery temperature and heating rate by a combined hyperelastic and viscoelastic model. Besides, 3D and 4D printing technologies are used to manufacture biomaterials structures due to rapid prototyping. Jia et al. proposed a self-expandable biodegradable vascular stent prepared by 3D printing. The compressed SMP stent showed excellent shape fixity [19]. Ge et al. presented multi-material SMP architectures with high resolution, which was up to a few microns [20]. This new 4D printing approach was applied to fabricate SMP stent with different geometries. Lin et al. summarized the progress of 3D and 4D printing technology of SMPs in biomedical field [21]. There has been a breakthrough of 4D printing technology in the personalized customization in the traditional medical field, which provided a new direction. The development of a shape memory polymer stent has acquired certain success. However, there are still many critical issues which should be solved before SMP stents can be used in clinical applications, particularly in terms of stent strength, buckling, and fatigue. In the complex loading environment of blood vessels, there are arbitrary or dynamic loadings from the pulsation of the heart or the displacement from body’s movement. Implanted stents also subjected to pressures of the surrounding vessels and blood flow. Therefore, it is necessary to discuss stability and buckling properties of a SMP stent. In particular, one should consider the strength of SMP stent due to the relatively small material modulus of SMP material. From a mechanics point of view, good flexibility for a tortuous vessel and a high radial strength supporting the plaque are the key attributes for successful SMP stent. Recent research showed that inadequate radial strength or stiffness had a strong influence on the clinical outcome such as stent dislocation [22,23]. There is the need to assess and design the radial strength of stent. Previous works also showed that the irregular mechanical interaction between stent and vessel can result in nutcracker [24]. One group tried to reduce the localized high stress by increasing the stent length, however, this would induce stent protrusion into the vascular lumen [25]. Consequently, optimization of the stent structure is required.

To solve the aforementioned issues, we proposed a new geometric model for SMP stent based on the metamaterial concept. Metamaterials [26,27,28] are artificial materials or structures which possess extraordinary physical properties. Mechanical metamaterials are one type of metamaterials which are man-made structures with superior mechanical properties [29], such as tunable stiffness, high strength, and negative Poisson’s ratio. Several studies explored the feasibility of auxetic structure applied in stent design. Ren et al. extended the latest methodology for generating 3D auxetic metamaterials to develop the metallic auxetic tubular structures [30]. The tubular structure exhibited auxetic behavior in both compression and tension, which enabled possible application in stents. Geng et al. proposed a chiral stent with two arrangements [31]. The tensile loading results showed that the chiral stent behaved negative Poisson ratio. Gatt et al. established an analytical model to describe the mechanical properties of a rotating square system [32]. Results showed that the prediction of Young’s moduli of infinitely sized systems was overestimated with finite-sized systems due to the edge effect. Gatt et al. also studied the suitability of three types arranged hexagonal honeycombs: non re-entrant, re-entrant, and hybrid honeycombs [33]. Simulation results exhibited possible migration of non re-entrant stent and higher risk of inflammation of hybrid stent due to shorter supporting length. Ali et al. validated an effective laser-cutting method to produce stent [34]. Results showed the mechanical behaviors of the produced stent with auxetic structure depended on the size and angle of individual units. The mechanical properties of mechanical metamaterials are mainly characterized by the unit cell of man-made structure, instead of material itself. The metamaterial especially the auxetic structure is showing a promising application in stent design. In this work, the new geometric model composed of series of modified auxetic unit cells (re-entrant structure) is developed for the SMP stent.

In the remainder of the paper, we first introduce the geometries of the expansion model of stent: vessel, plaque, and SMP stent in Section 2. Section 3 will present the constitutive models for materials used in expansion stent model: shape memory polymer, vessel, and plaque. Following the geometric models and constitutive models of expansion model, buckling analysis, and expansion process simulations are conducted using finite element method (FEM) in Section 4. To establish the effective assessment rule on the radial strength and stiffness of this type of SMP stent, an analytical solution on the SMP stent structure is accomplished in Section 5. The analytical results are compared with numerical results of FEM. Finally, the mechanical performances of the SMP stent with metamaterial are summarized.

## 2. Geometries of Expansion Model of SMP Stent with Vessel and Plaque 

### 2.1. Geometric Model of Vessel with Plaque 

**Figure 1:**
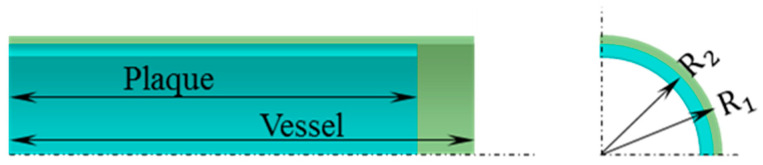
Geometric model of vessel and plaque (one-eighth).

In this work, an idealized model of a vessel with plaque is shown in Figure 1. For the simplicity, we assume the geometric model of the vessel with plaque as straight cylindrical tubes. Research showed a larger radius of a vessel exhibited a higher incidence of stroke, especially when the radius is larger than 4 mm [35]. Therefore, the outer radius of vessel symbolled with *R_1_* is set as 5 mm. The outer radius of inner plaque symbolled with *R_2_* is set with 4.9 mm and exhibited a thickness of 0.4 mm. The length of the vessel and plaque are 20 and 19 mm, separately. 

### 2.2. Geometric Model of Stent Based on Metamaterial

Research has shown that the structure design of vascular stents is the key to control the mechanical properties of stent and to improve clinical performance [36,37]. Diverse structures of vascular stent have been designed for various implant conditions. Gu et al. showed that the structure of the vessel stent had great importance on restenosis [38]. Therefore, the structure of the stent should be carefully designed for clinical applications.

**Figure 2:**
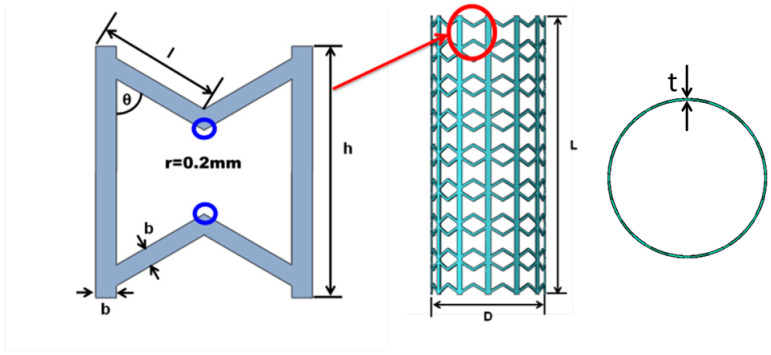
Unit cell and structure of the SMP stent.

Structures of traditional metal stents are usually selected with a small modulus with an aim to reduce the stiffness mismatch of stent and vessel and the restenosis after implantation. Differently, the modulus or strength of a SMP stent should be as large as possible due to the low stiffness at the level of ”MPa”. The radial strength should be large enough to expand the blocked vessel and stably support the vessel. Validated in Karnessis et al.’s work, a stent with re-entrant structure was demonstrated to have a better kinking property compared with the structure of positive Poisson’s ratio [39]. In this study, to improve the performance of a SMP stent, a modified re-entrant structure with the vertical side link mode is developed as the unit cell of the SMP stent (as shown in the Figure 2). This vertical side link mode was first introduced by Gandi and Olympio to form accordion cellular honeycombs [40].

To avoid the stress concentration, the blue circled corners are chamfered with the radius of 0.2 mm in Figure 2. The dimensional parameters defining the unit cell are shown in Table 1. l is the length of the slanted strut. θ is the angle between the slanted and vertical strut, which also characterizes the deformation space of the cell. b is the width of the strut. h is the height of the cell. t is the thickness of stent. D is the diameter of the stent and L is the height of the stent. Except for these parameters, two new parameters *N_C_* (circumferential number of unit cells) and *N_L_* (longitudinal number of unit cells) are introduced to describe the whole stent structure. We have

(1) $$\begin{array}{l} l\sin\theta =R\sin(\frac{\pi }{N_{C}})\\ L=N_{L}h \end{array}$$

where R=D/2, L is the height of SMP stent.

## 3. Constitutive Models of the Vessel, Plaque, and SMP 

To explore the mechanical performance of the expansion model of the stent, the constitutive relationships of the materails need to be determined first. The materials of the vessel and plaque are assumed incompressible and exhibit superelastic properties. Thus hyperelastic constitutive models are used to charaterize their materails properties. For the SMP stent material, a series of constitutive models are developed to describe the unique thermo-mechanical behavior [41,42]. Nevertheless, most constitutive models are confined with describing large deformation of SMPs or complex expressions [43,44,45,46]. In our work, a modified rheological model presented in our previous work [47] is adapted to analyze the thermo-mechanical behavior of SMP stent. This model uses a hyperelastic term to replace the elastic constant parameter and is valid to describe large deformation. The specific shape memory polymer used for stent design in this work is one kind of epoxy-based shape memory polymer (epoxy DA3) in [48].

### 3.1. Constitutive Models of the Vessel and Plaque

For the vessel and plaque, polynomial-form hyperelastic models proposed by Migliavacca et al. [49,50] are employed. The two models were shown to have good description of the mechanical performance of vessels and plaque. For the vessel, the constitutive equation is written as

(2) $$U=C_{10}\cdot (I_{1}-3)+C_{03}\cdot {\left(I_{2}-3\right)}^{3}.$$

where U is the strain energy density, *I*_1_ and *I*_2_ are the first and second Cauchy–Green tensor. The value of *C*10 and *C*03 are chosen to be 0.019513 and 0.02976 MPa, separately. For the plaque, the constitutive equation is expressed as

(3) $$U=C_{10}\cdot (I_{1}-3)+C_{02}\cdot {\left(I_{2}-3\right)}^{2}+C_{03}\cdot {\left(I_{2}-3\right)}^{3}.$$

The value of *C*_10_, *C*_02_, and *C*_03_ are chosen to be 0.04, 0.003, and 0.02976 MPa, respectively.

### 3.2. Constitutive Model of Shape Memory Polymer

**Figure 3:**
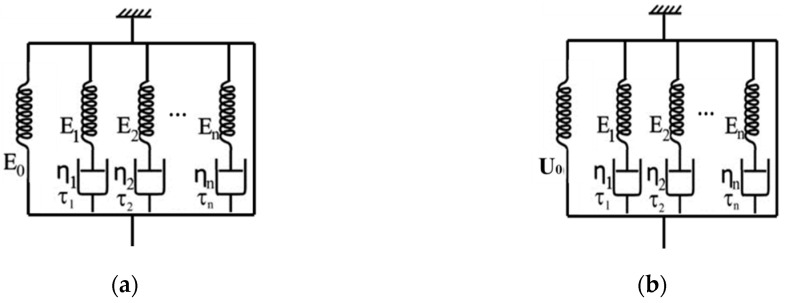
(**a**) Schematic of generalized Maxwell model; (**b**) Modified generalized Maxwell model.

A brief introduction of the modified constitutive model for the SMP stent is presented here. As shown in Figure 3, the model is composed a series of generalized Maxwell element. The elastic constant in Figure 3a is replaced by the hyperelastic term in Figure 3b, to enable large strains. 

The constitutive equation of the model shown in Figure 3a is derived as

(4) $$\sigma (t)=E_{0}\varepsilon_{0}+\varepsilon_{0}\sum_{i=1}^{n}E_{i}e^{-t/\tau_{i}}.$$

where σ(t) is the total stress, $E_{0}$ is the Young’s modulus of the elastic part, $\varepsilon_{0}$ is the strain of the model, t is the real time, $E_{i}$ and $\tau_{i}$ are the Young’s modulus and relaxation time of the Maxwell element, n is the number of Maxwell elements.

The generalized Maxwell model describes the viscoelasticity of the SMP. The experimental results in Diani et al. showed the model’s reliability under large deformation [48]. In the generalized Maxwell model, the relaxation modulus G(t) is written in the form of Prony series

(5) $$G(t)=G_{\infty }+\sum_{i=1}^{n}G_{i}\cdot e^{-t/\tau_{i}}.$$

where $G_{\infty }$ is the shear modulus at the infinite time and $G_{i}$ is the corresponding shear modulus of the Maxwell element.

The specific values of the above parameters are obtained from dynamic mechanical analysis experiments of the SMP by Fourier transformation. The relaxation moduli can be written by storage modulus and loss modulus

(6) $$G(\omega )=\sqrt{G_{s}{\left(\omega \right)}^{2}+G_{l}{\left(\omega \right)}^{2}}.$$

(7) $$G_{s}(\omega )=G_{0}+\sum_{i=1}^{n}\frac{G_{i}\tau_{i}^{2}\omega^{2}}{1+\tau_{i}^{2}\omega^{2}}.$$

(8) $$G_{l}(\omega )=\sum_{i=1}^{n}\frac{G_{i}\tau_{i}^{}\omega^{}}{1+\tau_{i}^{2}\omega^{2}}.$$

where $G_{s}(\omega )$ is the storage modulus and $G_{l}(\omega )$ is the loss modulus, ω is the test frequency of dynamic experiment. Here n is set as 12 which is enough to provide an exact description. From the experiments in [48], we can obtain that $G_{0}$ is 1.6 MPa. The specific values of $G_{i}$ and $\tau_{i}$ are listed in Table 2. 

In the model, the Williams–Landel–Ferry (WLF) equation shows the relationship of time and temperature, which is expressed as

(9) $$\mathrm{lg}(\alpha_{T})=\frac{-C_{1}\cdot (T-T_{r})}{C_{2}+T-T}.$$

where $\alpha_{T}$ is time-temperature superposition shifting factor, $C_{1}$ and $C_{2}$ are material constants and $T_{r}$ is the reference temperature. In this study we take the following values $C_{1}$ = 10.17, $C_{2}$ = 47.35 °C and $T_{r}$ = 50 °C.

The hyperelastic term is written in form of neo-Hookean equation

(10) $$U=C_{10}(I_{1}-3)+\frac{1}{D_{1}}(J_{el}-1).$$

where the first Cauchy–Green tensor. $J_{el}$ is the elastic volume strain. Note that $C_{10}=G\prime /2$,$D_{1}=2/K\prime$, where $G^{\prime }$ and $K^{\prime }$ are the initial shear modulus and bulk modulus at initial time. In this work, $K^{\prime }$ is 3.1 GPa. The coefficients $C_{10}$ and $D_{1}$ are taken as 393.964 and 0.0006452 MPa.

Implementing the geometrical models and constitutive equations using commercial software (ABAQUS 6.14), we can perform numerical simulations of mechanical behavior of the SMP stent.

## 4. Numerical Simulation for SMP Stent with Mechanical Metamaterial

As mentioned before, the stent is exposed to loadings from heart pulsation and blood flows [51,52], which could result in the stent’s migration and vessel restenosis [24,25]. To investigate the possible failure modes of the stent, a buckling analysis is performed. 

### 4.1. Buckling Analysis of SMP Stent

Here we simplify that the external loadings of stent including heart pulsation and blood flow can be modeled as radial pressure. Therefore, the buckling analysis is conducted under radial pressure. We assume there is no slippage between the vessel and the supporting stent. The movement along the axial direction of the stent is constrained at both ends. The buckling analysis is conducted at the stent’s recovery temperature (50 °C), at which the stent exhibits a soft state and is easier to go through failure. The buckling modes for the SMP stent with different radii are presented.

#### 4.1.1. Eigenvalue Analysis

Before nonlinear analysis of the stent’s cylindrical structure, a linear buckling (eigenvalue) analysis is carried out usin ABAQUS. To verify the feasibility of analysis, a mesh convergence is conducted first. The first three buckling modes are extracted (as shown in Table 3) with *Nc* = 8 type stent.

**Figure 4:**
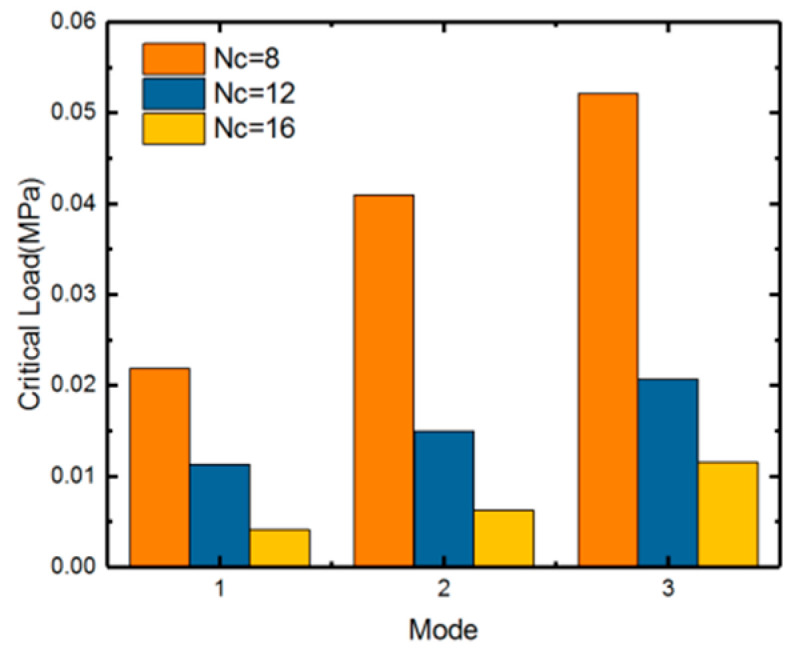
First three orders buckling eigenvalues of SMP stent with different numbers in circumferential direction: *Nc* = 8/12/16.

Table 3 shows the critical buckling loads of three different stent radii for the first three failure models. We can see that the critical load is larger with the smaller radius (smaller *Nc*), as shown in Figure 4. It could be explained that the stent of a smaller radius behaves a higher stiffness with same geometric unit cell.

#### 4.1.2. Nonlinear Buckling Analysis

**Figure 5:**
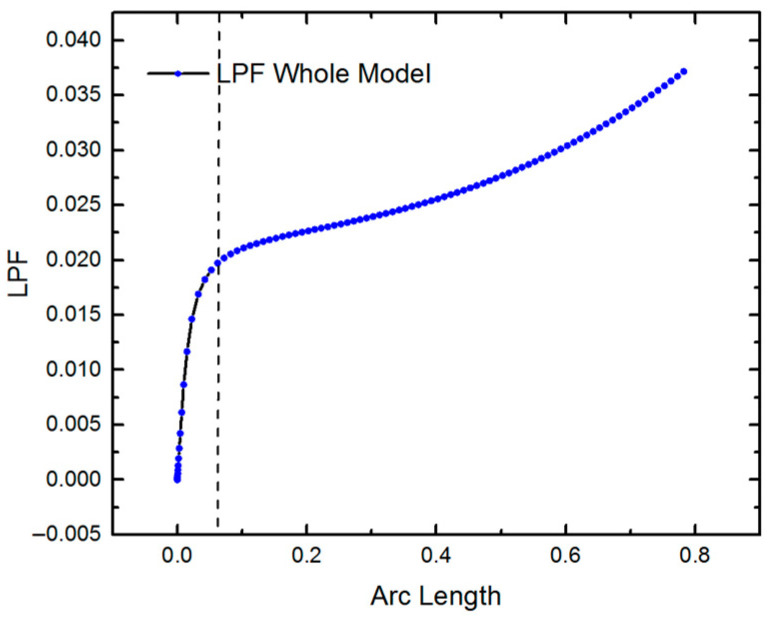
LPF curve of SMP stent.

Actually, the large deformation of structure during buckling often induces nonlinearity and thus the linear analysis is conservative. Here, we further conducted the nonlinear analysis of the SMP stents for *N_C_* = 8/12/16 using Riks’ method. The load is considered with 1 MPa. Figure 5 illustrates the buckling performance of the stent with *Nc* = 8. The curve shows a turning point approximately at the load proportionality factor (LPF) of 0.02, where the structure first buckles. The critical load is 1 × 0.02 = 0.02 MPa. The nonlinear analysis yields a smaller critical buckling load compared to that of the linear analysis, due to the nonlinear deformation and material nonlinearity of the SMP stent. Table 4 shows the static buckling load comparison for different values of radii (or different values of *Nc*). The results confirm that a stent with a larger radius buckles more easily. 

**Figure 6:**
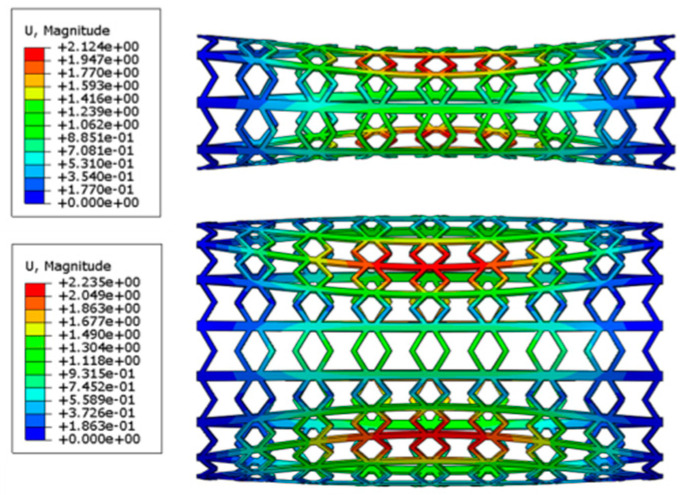
Buckling displacement amplitude of SMP stent with two different radiuses: 5.43 mm (*Nc* = 8) and 10.65 mm (*Nc* = 16).

The buckling displacement amplitude of the stents is also presented in Figure 6. At the same load of 1 MPa, the SMP stent with a larger radius shows a larger buckling displacement, which will result in greater damage to vessels.

### 4.2. Expansion of SMP Stent with Metamaterial in a Blocked Vessel

Applying the geometric models and constitutive models of the SMP stent in blocked vessel, the in vivo expansion performance of the SMP stent of metamaterial is investigated. The deformation process of the SMP stent is programmed as:

(1) Compress the stent to a smaller radius at a high temperature (*T* > *T*g);
(2) Cool the stent to temperature lower than *T*g;
(3) Release the load conducted on the stent;
(4) Deliver the stent to the target vessel;
(5) Stimuli the stent by heat, light, or magnetism;
(6) Remove the stimuli and cool the stent to body temperature.

**Figure 7:**
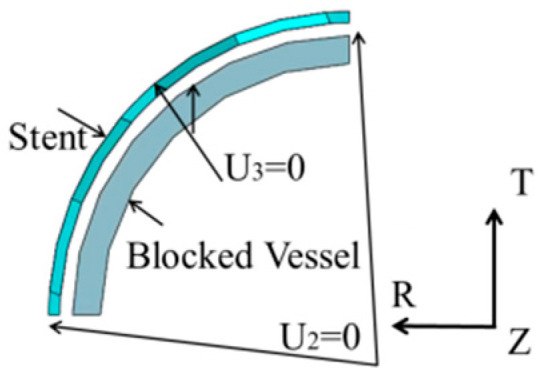
Loading conditions for the expansion model.

From the buckling modes shown in Table 3, a symmetric deformation is observed. Therefore, only one-eighth of the expansion model including the SMP stent and blocked vessel is studied. The symmetry loading conditions are used on the expansion model, as shown in Figure 7. A cylindrical coordinate system is set: *U*_1_ represents the radial displacement, *U*_2_ represents the rotational angle, and *U*_3_ represents the longitudinal stretch. The rotational angle of the two sides in the longitudinal direction of the expansion model is set to zero. The longitudinal displacement of one end of the expansion model is fixed to zero and the other end is free.

**Figure 8:**
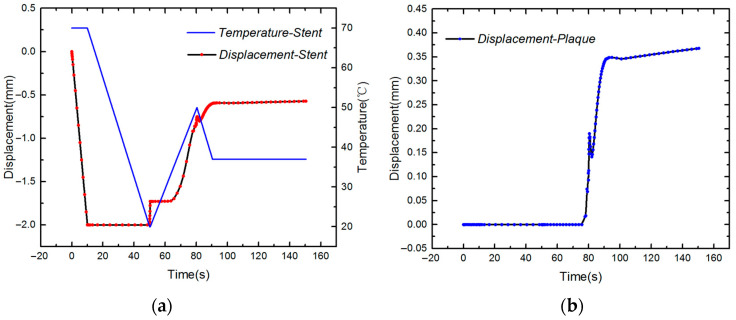
(**a**) Temperature and displacement of the SMP stent; (**b**) displacement of plaque.

Simulation results of the deformation of SMP stent and plaque can be seen from Figure 8. We can see that this stent successfully expands the plaque and reaches a stable displacement at the body temperature. A displacement fluctuation is observed during the last cooling step (6), presumably because the released recovery force of the SMP stent in step (5) is relocked during the cooling process and thus the small spring-back appears. On the other hand, as the temperature decreases, the strength of the stent increases and thus displacement of the stent continuously increases. As the temperature stabilizes at the body temperature, the displacements of the stent and plaque become steady.

To study the stretch of the SMP stent and plaque in the longitudinal direction, we collect the length of the stent and plaque before and after implantation. The longitudinal elongation ratio is defined as

(11) $$\alpha_{L}=\frac{L^{\prime }-L}{L}.$$

where L is the original length and $L^{\prime }$ is the deformed length. 

After the whole expansion process, we find a stretch of 0.635 mm of plaque and −0.0677 mm of the stent in the longitudinal direction. Thus, the longitudinal elongation ratios of the plaque and the stent are 3.3% and −0.35%, respectively. The stent almost has zero deformation in the longitudinal direction. This zero stretch reduces the interaction of the stent with the vessel and further reduces the vessel stenosis.

**Figure 9:**
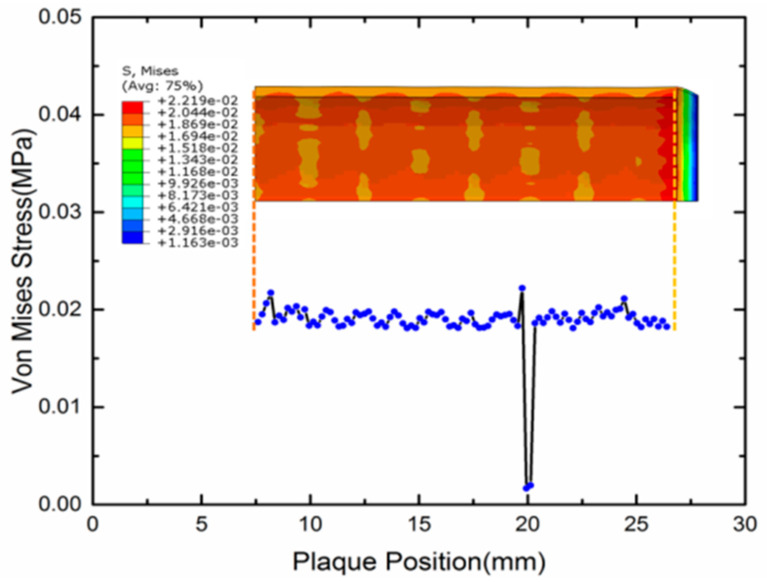
Von Mises stress contours of the plaque along the stent length.

**Figure 10:**
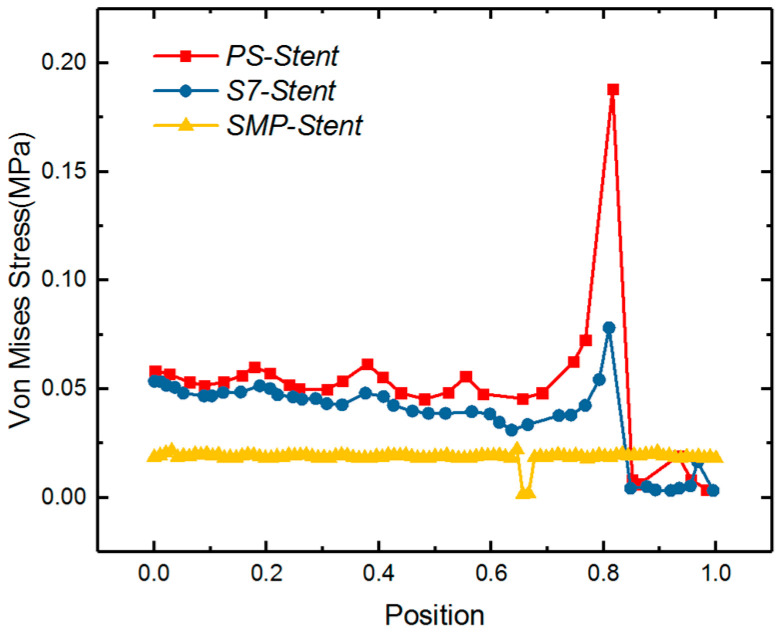
Von Mises stress comparison of length normalized vessel under expansion of three types of stent: balloon-expandable Palmaz–Schatz (Ps) stent, S670 stent [38], and SMP stent.

Research has shown that the stress of stented vessel can cause the restenosis of a vessel. Timmins et al. computationally and experimentally showed that the vascular stent implantation can introduce the increment of neointimal tissue [53]. Higher stress of the stented vessel was observed in a higher restenosis area [54]. Gu et al. also made a detailed finite element computation to present the relationship of stress and restenosis [38]. Their result showed that the excessive stretching of a vessel can result in proliferation of muscle cells, therefore, resulting restenosis of the vessel. Therefore, the stress level of vessels after a SMP stent implantation should be given much attention. In Figure 9, the stress distribution of the expanded vessel and plaque is depicted after the full expansion process. It can be observed that the stress along the plaque length direction is around 0.02 MPa. Normalizing the length of vessel, the von Mises stress of three different types of stents including the SMP stent is compared in Figure 10. We can see that the SMP stent exhibits a much smaller stress distribution and stress gradient. This indicates that the SMP stent of metamaterial could reduce the restenosis due to the low implanted stress. 

## 5. Analytical Model of Evaluating Radial Strength of SMP Stent

Radial strength which represents the ability to resist deformation under pressure is significant characteristic in development of vascular stents. Evidences [22,23] showed that inadequate radial strength or stiffness had strong influence on the clinical outcome such as stent dislocation. However, there is no standard equation definition for radial strength. Previous research analyzing the radial strength of stent concentrated on experiments [55,56] and numerical calculations [57], where radial strength or stiffness was often represented or quantified by the responsive radial force. There is no intrinsic description of the stent’s radial strength determined from geometry. In this work, an elementary theoretical analysis is conducted to assess the radial strength of the SMP stent.

We first derive the mechanical parameters of the unit cell and then develop the equations to the whole stent structure. The analytical results are validated against numerical results. Based on the equivalent modulus derivation, a preliminary radial strength assessment is demonstrated in the form of radial force. 

### 5.1. Mechanical Behavior of SMP Stent Unit Cell

**Figure 11:**
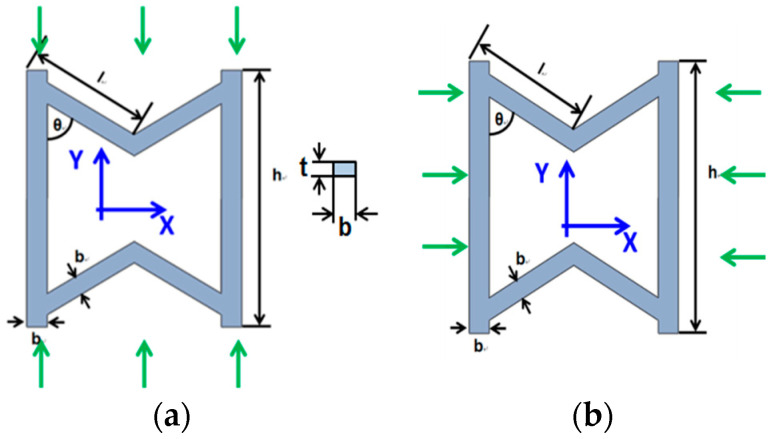
(**a**) Load force of stent unit cell in *x*-direction; (**b**) load force of stent unit cell in *x*-direction.

The SMP stent is arranged in a series of auxetic unit cell, which represents the mechanical performance of the whole structure. Masters et al. proposed that the global mechanical parameters of the whole tubular structure can be computed by assuming the struts behaving as fixed-guided beams or rods [58]. Considering the linking method of the unit cell, we presume that the deformation of unit cell is composed of stretch and bending. As shown in Figure 11, in the *y*-direction, the stretch dominates the mechanical behavior of the unit cell and in the *x*-direction this structure exhibits the most bending property. 

For modulus of the *y*-direction, it is assumed that the unit structure only deforms along the axial direction, and the two ends of inclined walls are fixed in the *x*-direction. The total stretch deformation becomes the sum of the vertical member stretch and deformation components of the slanted members in the *y*-direction. Thus, applying the stress of the *y*-direction $\sigma_{y}$, we write the total stretch as

(12) $$\varepsilon_{y}^{total}=\frac{\sigma_{y}\left(h+2l\cos^{2}\theta \right)l\sin\theta }{bhE_{s}}.$$

where $E_{s}$ is the modulus of stent material.

Then the stiffness of unite cell in Y direction can be written as

(13) $$E_{y}^{total}=\frac{\sigma_{y}}{\varepsilon_{y}^{total}}=\frac{E_{s}hb}{\left(h+2l\cos^{2}\theta \right)l\sin\theta }.$$

The strain in the direction *x* induced by the *y* direction force $\sigma_{y}$ is zero

(14) $$\varepsilon_{x}^{y}=0$$

and the Poisson ratio is written as

(15) $$\nu_{x}=-\frac{\varepsilon_{{}_{x}}^{y}}{\varepsilon_{{}_{y}}^{total}}=0.$$

Similarly, in the X direction, we assume that bending absolutely dominates the mechanical behavior of the unit cell. The deformation is determined by the bending of the slanted members. Applying the stress of the X direction $\sigma_{x}$, the total strain in the X direction can be expressed as

(16) $$\varepsilon_{x}^{total}=\frac{\sigma_{x}tl^{2}h\cos^{2}\theta }{24E_{s}I\sin\theta }.$$

where t is the thickness of unit cell and I is the moment of inertia and $I=\frac{b^{3}t}{12}$.

Then the modulus of the unit cell in the X direction is

(17) $$E_{x}^{total}=\frac{\sigma_{x}}{\varepsilon_{x}^{total}}=\frac{24E_{s}I\sin\theta }{tl^{2}h\cos^{2}\theta }.$$

At the vertical side link method, strain in the *y*-direction induced by the *x*-direction force is very small and can be treated with zero. Therefore, the deformation in the *y*-direction induced by $\sigma_{x}$ can be written as

(18) $$\varepsilon_{y}^{x}=0.$$

The corresponding Poisson ratio is

(19) $$\nu_{y}=0.$$

We also use the ABAQUS FE approach to calculate the homogenized mechanical properties of the unit cell [59]. Note that the effective length of the slanted edges should be modified as

(20) $$l_{eff}=l-\frac{b}{\sin\theta }.$$

and the $l_{eff}$ is used to replace l in Equations (12)–(19).

**Figure 12:**
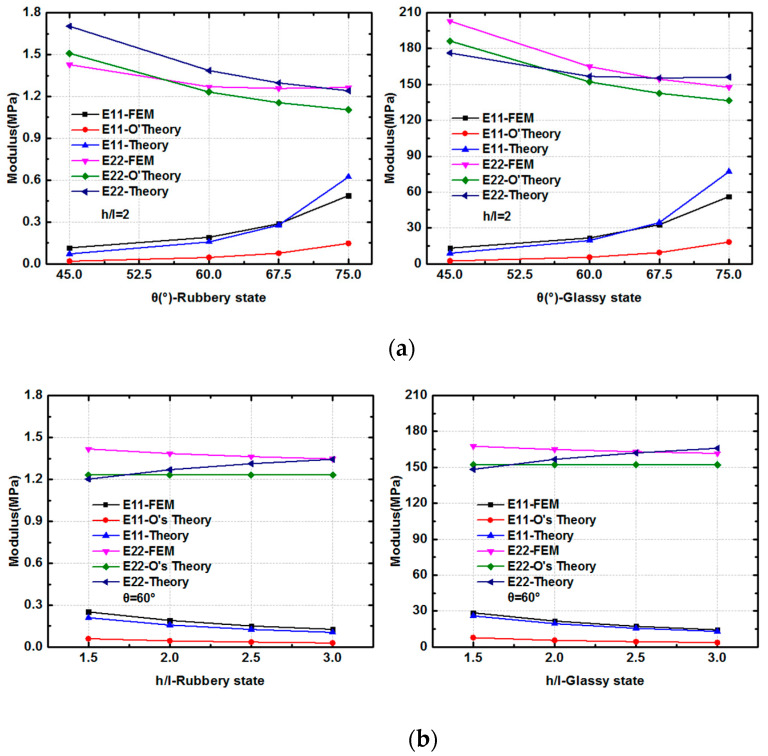
Stiffness of the unit cell in the *x*- and *y*-directions from two theoretical analyses and the FEM simulation (where E11 is the modulus in the *x*-direction, and E22 is the modulus in the *y*- direction): (**a**) Modulus at the rubbery state and glassy state with different θ; (**b**) Modulus at the rubbery state and glassy state with different hl.

For the SMP stent, there are two states in the process of expansion: the glassy state when delivering into vessel and the rubbery state when expanding the plaque. Therefore, the moduli at the two states are calculated. The FEM results are compared with the two analytical expressions: derived solutions in this paper and Olympio’s theory in [40]. From the results shown in Figure 12, we can find that at different material states and with different geometry parameters, good agreement is achieved between the two theories and finite element method for the description of Young’s modulus in Y direction. For Young’s modulus in X direction, both analytical solutions behave a correct trend while the theory in this work provides more accurate results. With a fixed width of inclined walls and vertical walls, varying the included angle θ and length ratio h/l, the analytical solutions could provide an effective description for the equivalent modulus at two directions.

**Figure 13:**
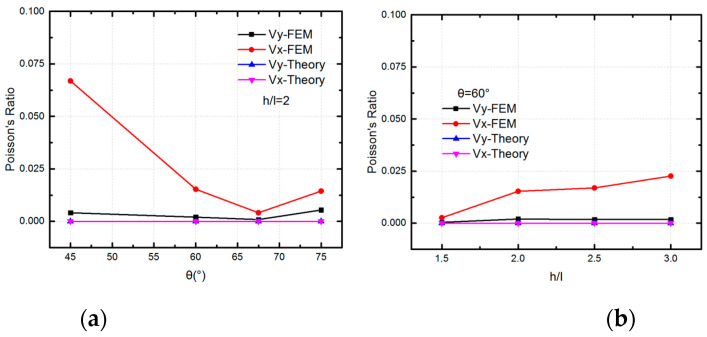
The FEM results and analytical results of Poisson ratios of the unit cell in the *x*- and *y*- directions (**a**) Poisson ratios with different θ; (**b**) Poisson ratios with different hl.

The analytical results of Poisson ratios of the unit cell in two directions at glassy state are compared with FEM results in Figure 13. There is a good agreement for $\nu_{y}$ with two results, which validates that the unit cell behaves a zero Poisson ratio in *y*-direction. However, the FEM results for $\nu_{x}$ shows that there is deformation of unit cell in the *x*-direction under $\sigma_{y}$. We can see that the deformation of inclined walls plays a more important role with a smaller included angle θ and a larger length ratio hl. This means the assumption for the deformation of inclined walls in the *x*- direction is over constrained. However, the maximum Poisson ratio is 0.0669 according to the FEM results with θ = 45°. That is to say the deformation of inclined walls in *x*-direction have a relatively small contribution to the deformation of unit cell. Thus, the Poisson ratio of $\nu_{x}$ is still treated with zero. 

### 5.2. Mechanical Properties of SMP Stent through Analytical Approach 

For the closed cylindrical structure, there are geometric relationships between the unit size and the whole structure size (as shown in Equation (1)).

As the longitudinal deformation is stretch, the longitudinal modulus of stent can be derived as

(21) $$E_{L}=\frac{\sigma_{L}}{\varepsilon_{L}}=\frac{E_{s}bL\sin(\theta )}{LR\sin(\frac{\pi }{N_{c}})\sin(\theta )+2N_{L}{\left(R\sin(\frac{\pi }{N_{c}})\cos\theta \right)}^{2}}.$$

where $\sigma_{L}$ is the longitudinal stress applied on the cross section of the stent and $\varepsilon_{L}$ is the corresponding longitudinal strain.

Similarly, the circumferential strain of the SMP stent is bending so the circumferential modulus of stent is

(22) $$E_{C}=\frac{\sigma_{C}}{\varepsilon_{C}}=\frac{24E_{s}I\sin\theta \tan^{2}\theta N_{L}}{t{\left(R\sin(\frac{\pi }{N_{C}})\right)}^{2}L}.$$

where $\sigma_{C}$ is the hoop stress applied on the hoop surface of stent and $\varepsilon_{C}$ is the corresponding circumferential strain.

**Figure 14:**
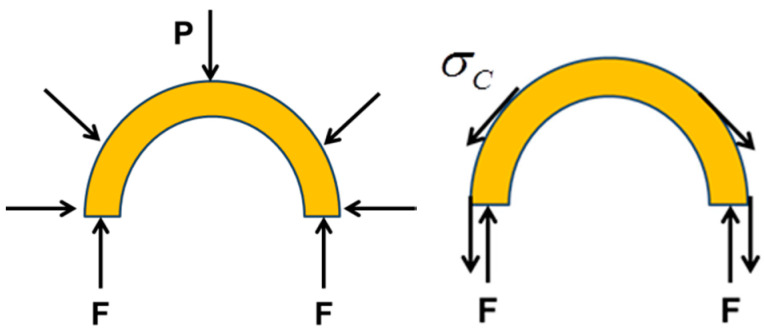
Illustration of force equilibrium relationship between radial direction and circumferential direction.

The radial modulus of the SMP stent is defined as

(23) $$E_{R}=\frac{P}{(D_{p}-D)/D}.$$

where P is the applied external pressure, D and $D_{p}$ are the original and deformed diameters of the stent. Using the force equilibrium as shown in Figure 14 (at the same deformation of radius), we can get

(24) $$\begin{array}{l} 2F=P\cdot D\\ F=\sigma_{C}\cdot t \end{array}$$

where F is the equilibrium force resulted from pressure P or hoop stress $\sigma_{C}$. 

Then we can obtain a relationship between $E_{R}$ and $E_{c}$

(25) $$E_{R}=\frac{P}{\varepsilon_{R}}=\frac{2t\sigma_{C}}{D\varepsilon_{C}}=\frac{24E_{s}I\sin\theta \tan^{2}\theta N_{L}}{R{\left(R\sin(\frac{\pi }{N_{C}})\right)}^{2}L}.$$

**Figure 15:**
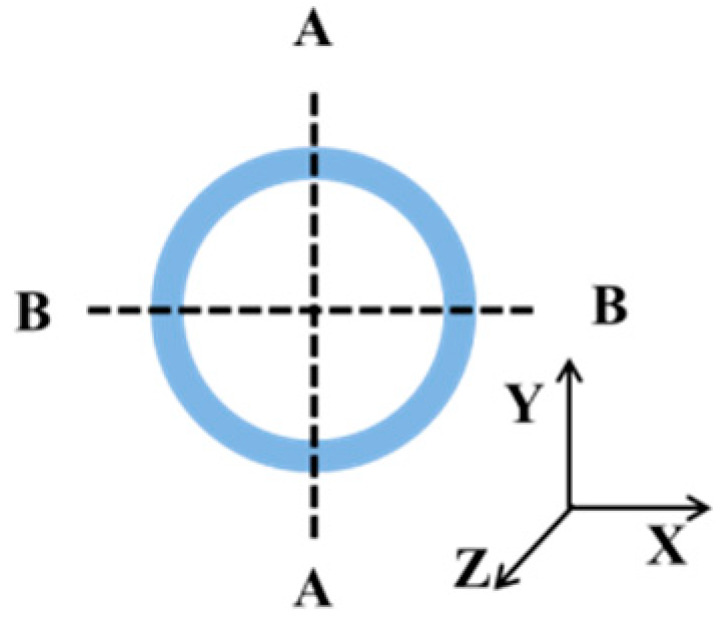
Illustration of the symmetric boundary conditions of stent.

To verify the efficiency of Equations (21–25), we compare the FEM results and analytical results for the whole stent structure. To obtain the numerical values of modulus in the radial and longitudinal directions, the symmetric boundary conditions of the numerical models are set, as shown in Figure 15. The displacement in the *x*-direction of the A-A section and the displacement in the *y*-direction of B-B section of stent are restricted. Applying a same displacement on two ends of the stent, the numerical value of the longitudinal modulus can be obtained by the corresponding stress and strain. Similarly, the numerical value of radial modulus can be obtained by applying a certain pressure on the outer surface of the stent.

**Figure 16:**
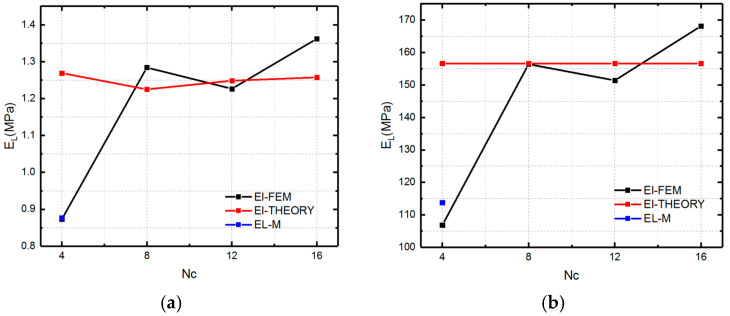
Modulus (El-FEM representing the simulation results of longitudinal modulus of SMP stent, El-THEORY representing the theoretical results of longitudinal modulus of stent and EL-M representing the modified result of longitudinal modulus of stent for *Nc =* 4) of the SMP stent in the longitudinal direction at two states: (**a**) rubbery state; (**b**) glassy state.

We first compare the theoretical and numerical values of the longitudinal modulus at two material states, which are shown in Figure 16. 

Figure 16 presents the numerical and theoretical results of longitudinal modulus of SMP stent which are symbolled with El-FEM and El- THEORY. From Figure 16, it can be found that there is an obvious discrepancy between FEM simulation and the analytical analysis for *Nc =* 4 stent. This could be explained by the fact that when the circumferential number is too small, to reach enough compressed strain, the bending deformation starts to dominate the deformation behavior of the stent. Therefore, a modified coefficient (0.88, 0.12) representing the bending and stretch proportion is added to give a more accurate description of the SMP stent which is symbolled with EL-M (as shown in Figure 16a,b). The coefficients are effective for the longitudinal modulus of SMP at both rubbery and glassy states. 

**Figure 17:**
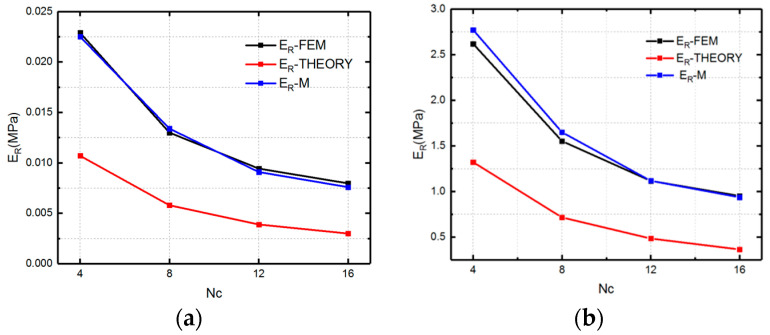
Elastic modulus of the auxetic stent in the radial direction at two material states: (**a**) rubbery state (high temperature); (**b**) glassy state (low temperature).

Figure 17 shows the comparison of the numerical results and theoretical results for the radial modulus. To improve the accuracy of the equations, the deformation mechanism of the stent is optimized. Considering the difference of the closure of stent and the infinity of the unit cell, the circumferential deformation is reconsidered as a combination of bending and stretch. In other words, the slanted members of the stent bended and compressed simultaneously when the stent is compressed. Therefore, the radial strain is written as

(26) $$\varepsilon_{r}=\alpha \varepsilon_{br}+(1-\alpha )\varepsilon_{sr}.$$

where $\varepsilon_{br}$ and $\varepsilon_{sr}$ are the radial components of bending and stretch deformation of the slanted members, respectively, and α is the bending proportion in the total strain.

Assuming the slanted members only stretch when loaded $\sigma_{x}$, the bending deformation in the *x*-direction of unit cell is derived as

(27) $$\varepsilon_{x}=\frac{\sigma_{x}h\sin\theta }{2bE_{s}}.$$

Extending the deformation to the whole structure of stent, the radial modulus of stent can be written as

(28) ER=tR(αt(RsinπNC)2Lcos2θ24EsNLIsin3θ+(1−α)LsinθNL2bEs).

The modified theory fits the FEM results well. The specific values of α are shown in Table 5. It can be seen that the bending deformation play a more important role for the stent with the smaller *Nc*.

As shown in Figure 17, a good agreement between the numerical and theoretical results is obtained. That is to say, this analytical solution can successfully describe the mechanical properties of the metamaterial-based SMP stent. Tunable modulus of SMP stent can be achieved by changing the heating temperature [13] as well as selecting different geometric parameters. Thus, both the numerical and theoretical results show that the stent with a smaller *Nc* exhibits a larger modulus and thus a larger radial deformation resistivity. In other words, the SMP stent with smaller radius could provide larger radial force under the same expansion displacement requirement. 

Now we try to define the radial strength, which is the important indicator of stent’s performance. The radial strength of a stent represents its ability to resist the deformation induced by outer pressure. As there is not a standard definition or equation for the radial strength of stents, in this study, we use the radial force (RF), which comes from the applied pressure, to define the radial strength.

The definition of the radial force can be written as

(29) $$F_{R}=P\cdot S.$$

where P is the loaded radial pressure, S is the radial area, S=2πR.

Then we can obtain

(30) F=ER⋅εR⋅2π⋅R=2πtR(αt(RsinπNC)2Lcos2θ24EsNLIsin3θ+(1−α)LsinθNL2bEs)⋅(R′−R).

where $R^{\prime }$ is the programmed deformation radius which is smaller than stent’s radius (R). 

In practice, the size of a blocked vessel is predetermined, and therefore, the programmed deformation radius for a stent with different geometries is the same. From Equation (30), keeping the same geometric unit cells, we can find that if a SMP stent is deformed to a same target radius, the stent with the bigger radius has higher radial force. This is because the stent with a bigger radius undergoes a larger deformation and thus accumulates more radial force. 

In addition, we should notice that there is an ultimate stress which is limited by the deformation space (θ) of the unit cell. As mentioned in Equation (26), we give the definition of the circumferential strain, for which the largest strain is $\varepsilon_{u}=\frac{l_{eff}\cdot \sin\theta \cdot N_{C}}{2\pi R}$. Then we can obtain a maximum circumferential stress and ultimate radial force (URF) as

(31) $$\sigma_{u}=E_{c}\varepsilon_{u}.$$

(32) $$F_{u}=4\cdot \pi \cdot t\cdot \sigma_{u}.$$

The F which is smaller than the maximum compressed force $F_{u}$ is effective. For example, with the same geometric unit cell (*h* = 4.8 mm, *l* = 2.4 mm and θ=60°), we find that if we compress the stent with radius 8.03 mm (*Nc =* 12) to radius 4.5 mm (smaller than that of the plaque), the radial force would be $2.08\times 10^{-4}N$, while the actual limited force is $1.82\times 10^{-4}N$ from Equation (32). Therefore, a stent with this radius could not reach the expansion requirement. Furthermore, we discuss the practicability of SMP stent with a fixed radius of 8.03 mm. The difference of the URF and RF of an SMP stent is written as

(33) $$D_{F}=F-F_{u}=2\pi R\cdot E_{R}\cdot (\varepsilon_{u}-\varepsilon_{R}).$$

The SMP stent is of practicability when $D_{F}$ is larger than zero. There is a positive correlation of $D_{F}$ and $\left(\varepsilon_{u}-\varepsilon_{R}\right)$, when $\left(\varepsilon_{u}-\varepsilon_{R}\right)$ can be normalized with parameters of circumferential numbers $N_{C}$ and the programmed radius $R^{\prime }$. The difference of targeted strain and ultimate strain is derived as

(34) $$\varepsilon_{u}-\varepsilon_{R}=\frac{(R\cdot \sin(\frac{\pi }{N_{C}})-b)\cdot N_{C}}{2\pi R}-\frac{\left(R-R^{\prime }\right)}{R}.$$

**Figure 18:**
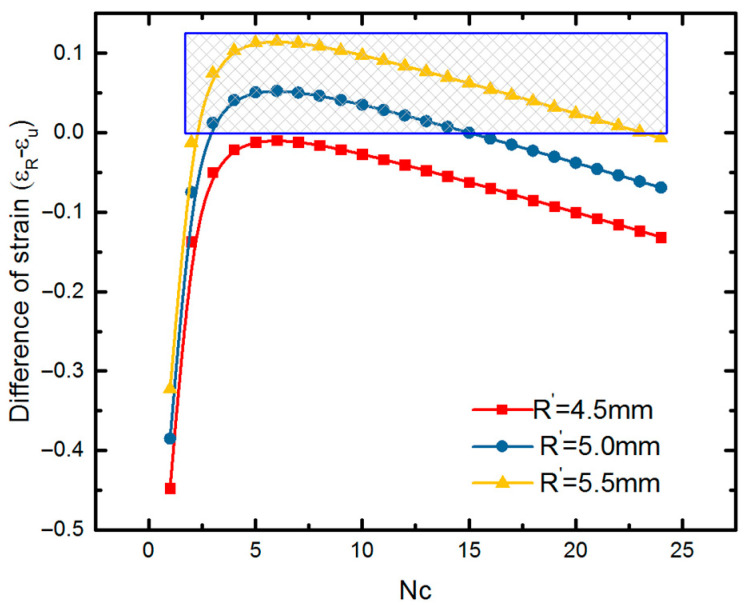
Difference of required strain and ultimate strain of the SMP stent with a radius for different programmed radii.

**Figure 19:**
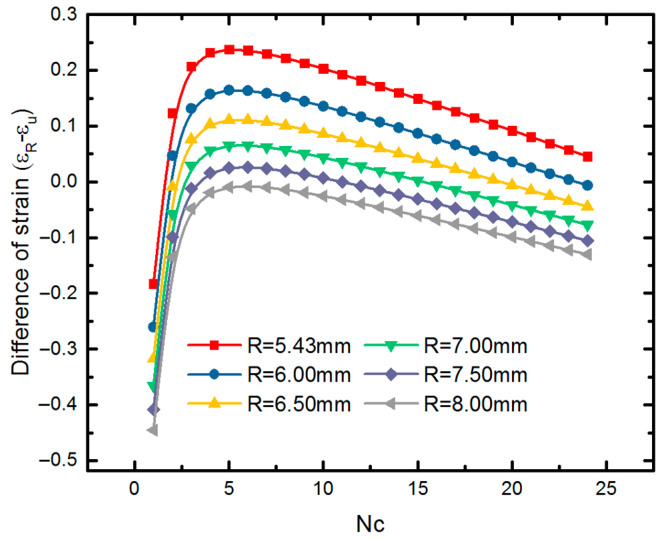
Difference of required strain and ultimate strain of SMP stent with different radii at the same programmed radius.

From Figure 18, we can find that for targeted radius of 4.5 mm in this work, a SMP stent with radius of 8.03 mm is unable to expand the blocked vessel due to its inadequate deformation. If the radius of vessel blockage is larger, such as 5.0 mm and 5.5 mm shown in Figure 18, then a stent with radius of 8.03 mm is effective with different circumferential numbers. The region marked with blue box represents the field where SMP stent is able to reach the required compression displacement. A maximum deformation space is found at *Nc =* 6. Similarly, we discuss the effect of radius on the deformation space of a SMP stent (as shown in Figure 19). It can be seen that a smaller radius provides a larger deformation space at the same circumferential number as well providing more permutations for stent design. 

This analytical solution reveals that the radial force is dependent on the radius and deformation space of the stent. Notably, the number of the periodic unit cell in the circumferential direction has a great effect on the deformation ability of the stent. Figure 19 shows that the stent obtains the largest deformation space when *Nc* reaches to the value of five or six. If the symmetry of the stent is considered, *Nc* should be six. From point of view of the radial force, the stent radius should be selected as the largest possible. However, from the point of view of buckling properties, a SMP stent with a smaller radius is preferred. Therefore, there is comprehensive consideration in selecting the stent radius. Results show that a tunable radial force of an SMP stent can be obtained by varying the radius of the stent and periodic circumferential numbers of the stent unit cell.

As the loading environment of a blocked vessel is complex and dynamically changing, the proposed analytical approach could be improved in future, to consider more factors such as the vessel–stent interaction, the wall shear force induced by blood flow. Nevertheless, our analytical description can already be used to provide some primitive guidelines for the design and assessment of the SMP stents.

## 6. Conclusions

For SMP stents, the self-expanding method makes a less harmful expansion for vessel plaque, while the strength should be carefully programmed to provide enough radial support force. In this study, a new geometry model for the SMP stent of metamaterial was proposed. This geometric model can reduce the axial stretch in length during expansion, due to the zero Poisson’s ratio. The simulation results revealed that the SMP stent has a comparatively small stress and stress gradient distribution of the expanded plaque. Therefore, we can say this geometric model of the SMP stent is effective to reduce the restenosis. The buckling analysis gave possible failure modes of the SMP stent under the complex mechanical environment of a blood vessel and indicated a smaller radius was superior to a stable expansion. Furthermore, a preliminary theory to assess the radial strength of the SMP stent was presented. The good agreements between the numerical and analytical results are found. We also provided a tunable radial strength of the SMP stent by changing the geometric parameters. In conclusion, this new geometric model based on metamaterial for the SMP stent is very promising in supporting a blocked blood vessel. The proposed analytical solution to evaluate the radial strength of the stent can also be used to provide guidelines for the design of the SMP stent structure.

## Figures and Tables

**Table 1:** Geometric parameters of the SMP stent (for *Nc* = 12, *N_L_* = 8)

*l* (mm)	θ (℃)	*b* (mm)	*h* (mm)	*D* (mm)	*L* (mm)	*t* (mm)
2.4	60	0.4	4.8	7.939	38.4	0.2

**Table 2:** Parameters of constitutive model of SMP: shear moduli and associated relaxation times

$G_{i}\left(Pa\right)$	$0.1476\times 10^{9},\;0.1756\times 10^{9},\;0.2025\times 10^{9},\;0.1775\times 10^{9},\;0.6802\times 10^{8},\;0.1139\times 10^{8},\;0.2264\times 10^{7},\;0.8132\times 10^{6},\;0.4020\times 10^{6},\;0.1760\times 10^{6},\;0.5056\times 10^{5},\;0.1265\times 10^{5}$
$\tau_{i}\left(s\right)$	$0.3031\times 10{\;}^{-4},\;0.1721\times 10^{-3},\;0.9768\times 10^{-2},\;0.5545\times 10^{-2},\;0.3147\times 10^{-1},\;0.1787,\;0.1014\times 10^{1},\;0.5757\times 10^{1},\;0.3268\times 10^{2},\;0.1855\times 10^{3},\;0.1053\times 10^{4},\;0.5977\times 10^{4}$

**Table 3:** The first three buckling modes of SMP stent with *Nc* = 8

Mode 1	Mode 2	Mode 3
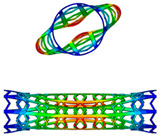	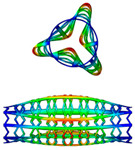	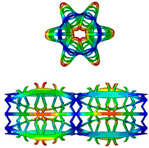

**Table 4:** First order static buckling load of SMP stents with different values of *Nc.*

*Nc*	8	12	16
Linear result (MPa)	0.025153	0.015455	0.00876847
Nonlinear Result (MPa)	0.02	0.012	0.0076

**Table 5:** Bending proportion of radial strain of the stent.

*Nc*	4	8	12	16
α	0.3	0.35	0.35	0.4
